# Opening a new window for targeted biopsy of biliary lesions: tapered hood–assisted cholangioscopy

**DOI:** 10.1055/a-2980-7483

**Published:** 2026-10-28

**Authors:** Takeshi Ogura, Masahiro Yamamura, Kimi Bessho, Junichi Nakamura, Hiroki Nishikawa

**Affiliations:** 1 Pancreatobiliary Advanced Medical Center 38588 Osaka Medical and Pharmaceutical University HospitalTakatsukiOsaka PrefectureJapan; 2 Endoscopy Center 38588 Osaka Medical and Pharmaceutical University HospitalTakatsukiOsaka PrefectureJapan; 3 2nd Department of Internal Medicine 13010 Osaka Medical and Pharmaceutical UniversityTakatsukiOsaka PrefectureJapan

Standard tissue-acquisition methods for diagnosing biliary strictures include brush cytology and forceps biopsy during endoscopic retrograde cholangiopancreatography (ERCP). 1​ For indeterminate biliary strictures, peroral cholangioscopy (POCS) offers several advantages, including the direct visualization of the bile duct and targeted biopsy under direct vision. However, lesions located on the lateral or inferior wall of the bile duct can be difficult to biopsy because the forceps approach them tangentially and may slip off the lesion, resulting in inadequate tissue acquisition. Moreover, the trajectory of the biopsy forceps may not align appropriately with the target lesion. A dedicated tapered hood for cholangioscopy has recently become available. This hood may overcome these limitations by centering the biopsy forceps within the endoscopic field and enabling the lesion to be stabilized by pressing the hood against it, thereby reducing forceps slippage during tissue acquisition. Herein, we describe technical tips for targeted biopsy using tapered hood–assisted POCS. A 49-year-old man underwent ERCP for tissue diagnosis of suspected cholangiocarcinoma. After successful guidewire cannulation, cholangiography showed a tight hilar biliary stricture ( Fig. 1 ). A dedicated tapered hood was therefore attached to a cholangioscope ( Fig. 2 ). The cholangioscope was easily advanced through the stricture to the hilar lesion because of the bougie-like dilating effect of the tapered hood ( Fig. 3 ). Targeted biopsy was performed on a lesion at the 6 o’clock position. With the hood pressed against the lesion to stabilize it and the biopsy forceps emerging through the center of the hood, tissue acquisition was easily performed ( Fig. 4 ). A lesion at the 3 o’clock position was similarly biopsied without difficulty ( Fig. 5 and Video 1 ). Histopathological examination of the biopsy specimens confirmed cholangiocarcinoma, and the patient subsequently received systemic chemotherapy. In conclusion, tapered hood–assisted POCS may facilitate advancement across a tight biliary stricture and targeted biopsy of difficult-to-access lesions by providing a bougie-like dilating effect, centering the biopsy forceps, and stabilizing the target lesion. Further studies are warranted to evaluate the safety and clinical utility of this technique.

**Fig. 1:**
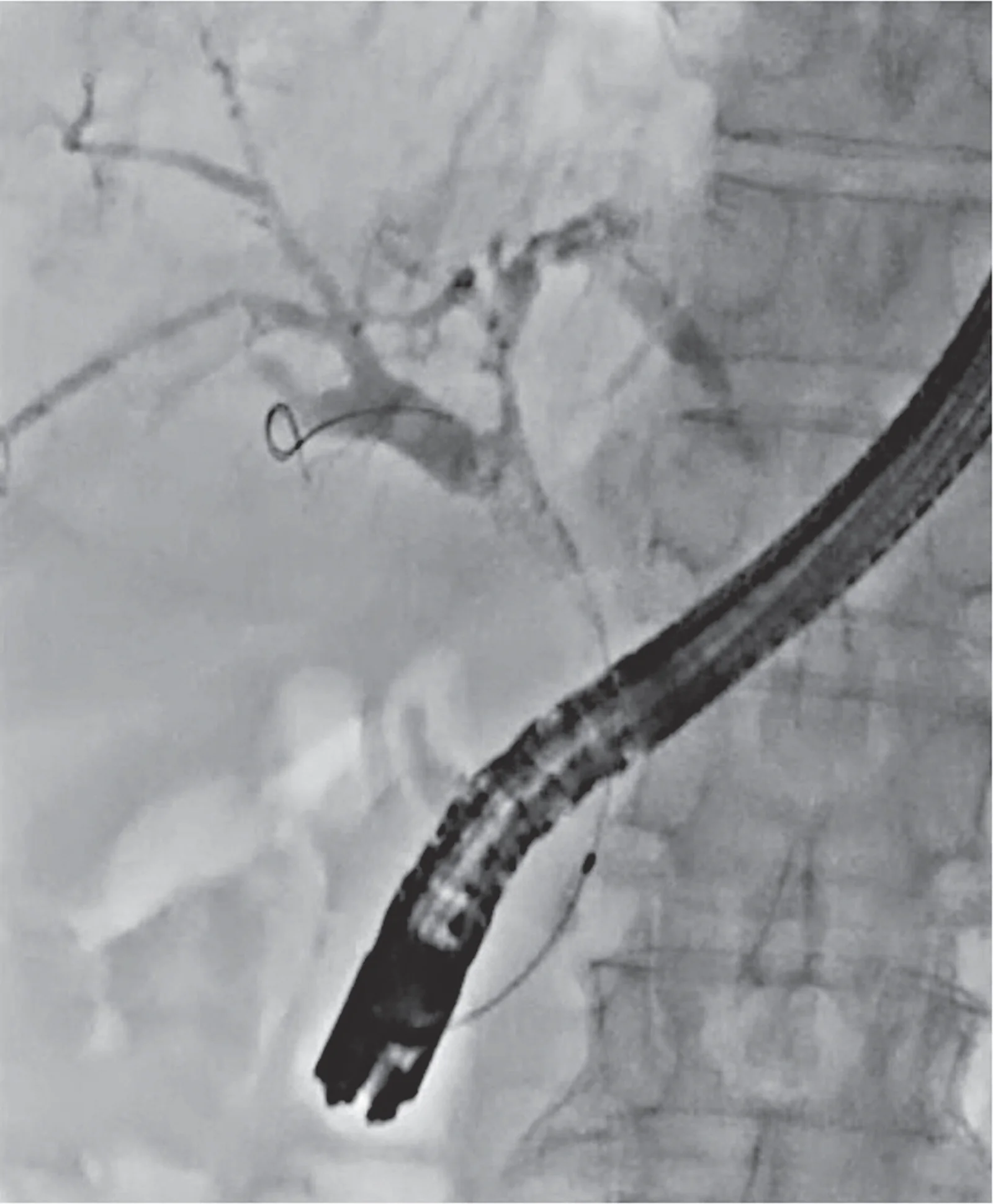
A bile duct stricture is observed.

**Fig. 2:**
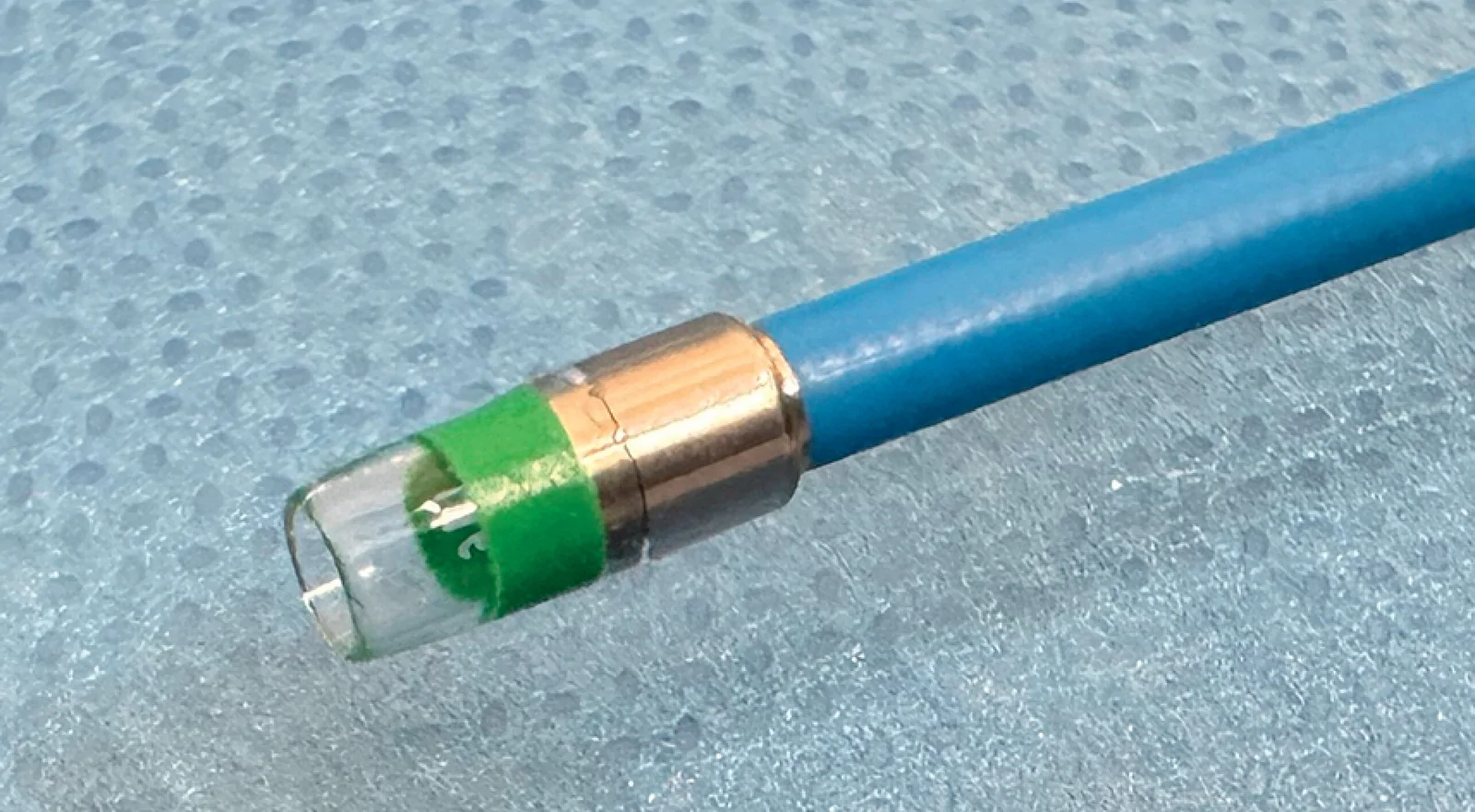
A tapered hood is attached to the cholangioscope.

**Fig. 3:**
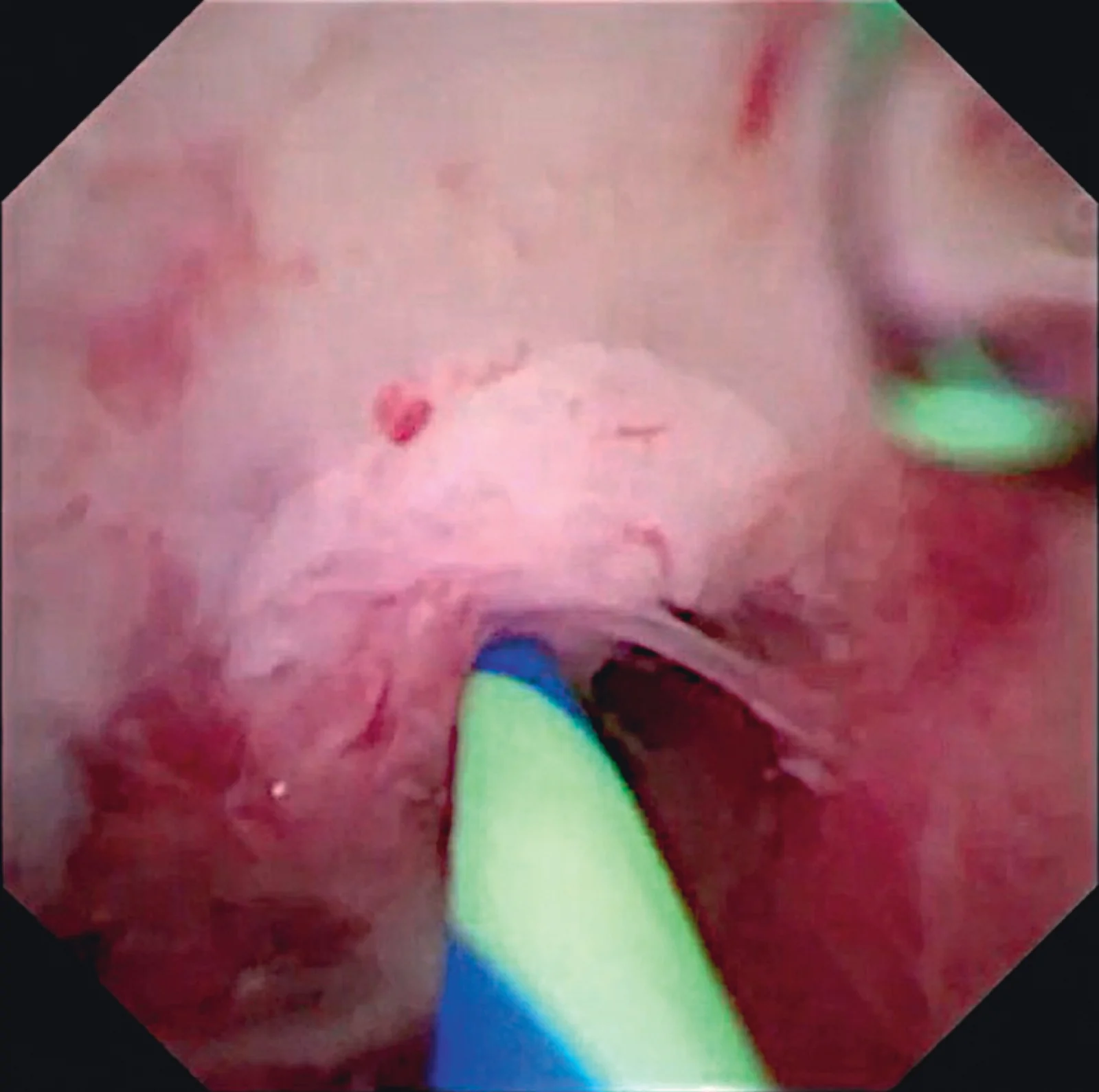
The cholangioscope is easily advanced through the stricture to the hilar lesion, presumably because of the bougie-like dilating effect of the tapered hood.

**Fig 4:**
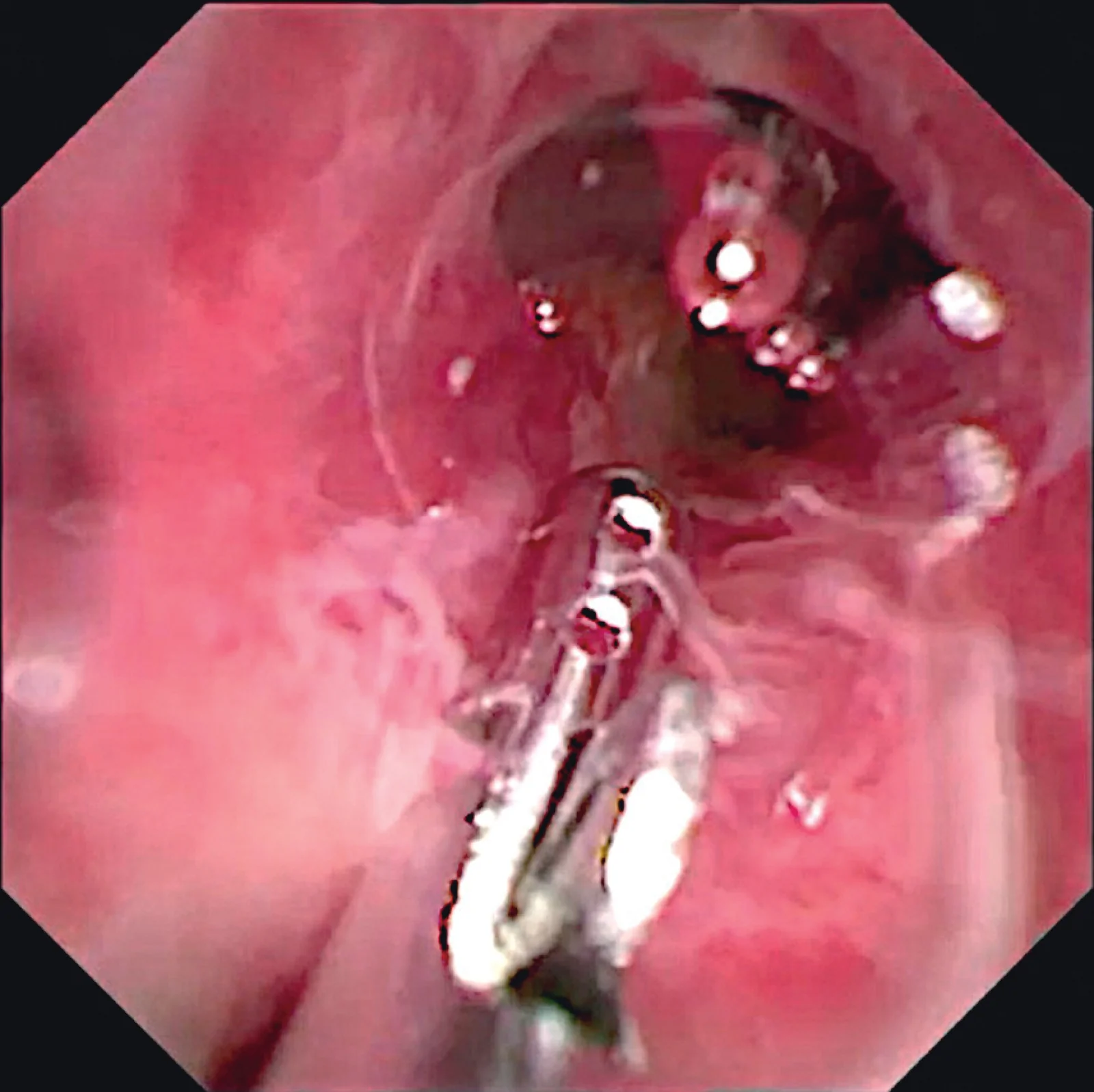
Targeted biopsy is performed on a lesion at the 6 o’clock position.

**Fig 5:**
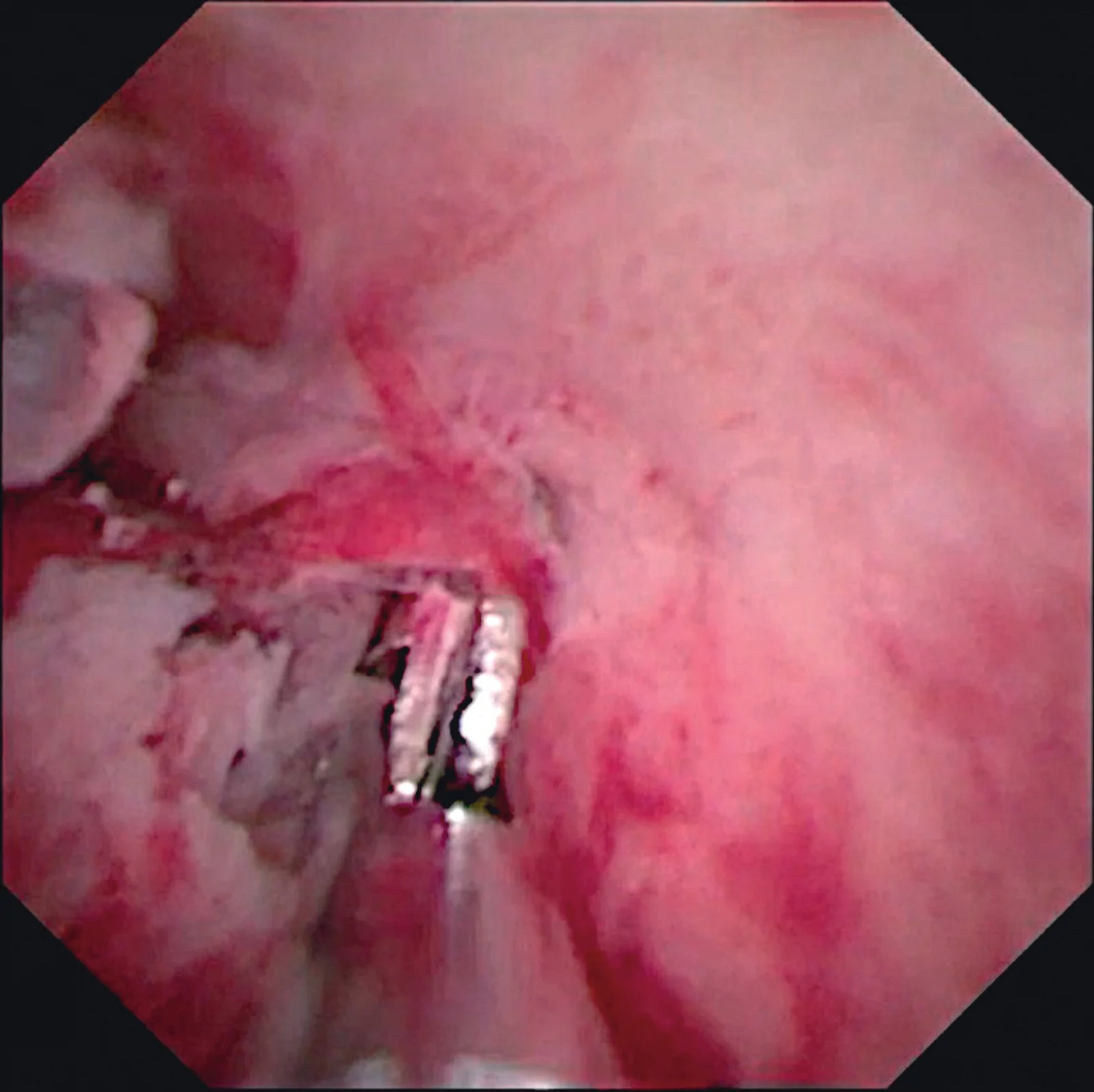
A lesion at the 3 o’clock position is similarly biopsied without difficulty.

**Video 1** A tapered hood is attached to the cholangioscope. URI: .


Endoscopy_UCTN_Code_TTT_1AR_2AL

## Declaration of GenAI Use

The authors confirm that no generative artificial intelligence was used in the preparation of this manuscript.
